# Functional and Structural Properties of Type V Collagen from the Skin of the Shortbill Spearfish (*Tetrapturus angustirostris*)

**DOI:** 10.3390/molecules29112518

**Published:** 2024-05-27

**Authors:** Qiuyu Han, Tomoyuki Koyama, Shugo Watabe, Shoichiro Ishizaki

**Affiliations:** 1 Graduate School of Marine Science and Technology, Tokyo University of Marine Science and Technology, 4-5-7 Konan, Minato, Tokyo 108-8477, Japan; fancy_hqy@163.com (Q.H.);; 2 School of Marine Biosciences, Kitasato University, Minami, Sagamihara 252-0373, Kanagawa, Japan

**Keywords:** type V collagen, cDNA cloning, shortbill spearfish, AlphaFold2, bioactivity

## Abstract

Type V collagen is considered to be a crucial minor collagen in fish skin with unique physiological functions. In this research, the cDNAs of three procollagens (Tacol5a1, Tacol5a2, and Tacol5a3) in type V collagen were cloned from the skin of shortbill spearfish (*Tetrapturus angustirostris*). The open reading frames (ORFs) of Tacol5a1, Tacol5a2, and Tacol5a3 contained 5991, 4485, and 5607 bps, respectively, encoding 1997, 1495, and 1869 amino acid residues. Each of the deduced amino acid sequences of procollagens contained a signal peptide and a fibrillar collagen C-terminal domain (COLFI). A conserved thrombospondin-like N-terminal domain (TSPN) was found at the N-terminus of Tacol5a1 and 5a3 procollagens, whereas a von Willebrand factor (VWC) was found at the N-terminus of Tacol5a2 procollagen. Tacol5a1, Tacol5a2, and Tacol5a3 had their theoretical isoelectric points of 5.06, 6.75, and 5.76, respectively, and predicted molecular weights of 198,435.60, 145,058.48, and 189,171.18, respectively. The phylogenetic tree analysis revealed that Tacol5a1 of shortbill spearfish clustered with that of yellow perch (*Perca flavescens*) instead of broadbill swordfish (*Xiphias gladius*). In addition, type V collagen was extracted from the shortbill spearfish skin. The *in silico* method demonstrated that shortbill spearfish type V collagen has a high potential for angiotensin-converting enzyme (ACE) inhibition activity (79.50%), dipeptidyl peptidase IV inhibition (74.91%) activity, and antithrombotic activity (46.83%). The structural clarification and possible functional investigation in this study provide the foundation for the applications of exogenous type V collagen derived from fish sources.

## 1. Introduction

Collagen, an integral fibrous protein, is ubiquitously found across vertebrate organs, notably enhancing skin and bone tissue functionality [1]. As of now, researchers have identified 29 distinct collagen types [2]. Differentiated into fibrillar and non-fibrillar categories based on their structural and functional roles. Type II, III, IV, and IX collagen have been extensively studied in osteoarthropathies [3,4], Ehlers–Danlos syndrome [5], cardiomyopathic fibrosis [6], ocular diseases [7], Alport syndrome [8,9], and multiple epiphyseal dysplasia [10], among other pathological disorders. Type V collagen, a minor fibrillar collagen subtype isolated through pepsin hydrolysis, plays a critical role in the regulation of procollagen fiber formation within connective tissues expressing type I collagen [11,12]. Comprising three subunits or α chains with similar amino acid sequences, fibrillar collagen is pivotal in tissue architecture and integrity. Initially secreted by cells like fibroblasts and chondrocytes, procollagen undergoes extracellular modifications, including covalent cross-linking and cleavage of peptide bonds, facilitating the transition to mature collagen fibers [13]. In fibrillar collagens, procollagen includes N-terminal and C-terminal prepeptides, N-terminal and C-terminal telopeptides, and a triple-helical structural domain containing the Gly-X-Y repeater. Typically, the X position is proline, and the Y is hydroxyproline.

In fibrous tissues, the composition and ratio of procollagen chains within type V collagen significantly influence health, pathological states, and fibrotic conditions. Markedly increased levels of type V collagen have been identified in tissues compromised by conditions such as cancer, granulation tissue, atherosclerosis, and fibrosis [14]. Mouse embryos lacking the pre-α1(V) chain show early fatality, highlighting the chain’s essential role in development [15]. Notably, mutations in the Col5a1 or Col5a2 genes, which encode the α1(V) and α2(V) procollagen chains, respectively, were observed in half of the individuals diagnosed with classical Ehlers–Danlos syndrome (EDS) [16]. Furthermore, elevated expression of the Col5a3 gene, responsible for the α3(V) procollagen chain, has been inversely associated with gastric cancer survival rates [17]. Nevertheless, mice genetically modified to lack the α3(V) gene did not show pronounced EDS-like symptoms, but displayed characteristics associated with obesity, including reduced subcutaneous skin layers. This suggests that the α3(V) chain interacts directly with cellular surface components, influencing islet cell functionality and differentiation, potentially leading to insulin resistance [18].

The shortbill spearfish (*Tetrapturus angustirostris*), a member of the Pacific billfish family, is recognized as one of the largest bony fishes in oceans [19]. As highly migratory apex predators exhibiting solitary behaviors, these fish present substantial research challenges [20]. The harvest of Pacific billfish, including shortbill spearfish, often occurs as a secondary catch in fisheries targeting more valuable species. Despite an overall increase in Pacific billfish catches since 1990, the annual catch rates for shortbill spearfish have consistently declined [21]. Unlike other billfish species such as broad swordfish (*Xiphias gladius*) [19,22], current research on shortbill spearfish is limited.

Marine organisms are esteemed as superior sources of collagen, attributed to their minimal risk of transmitting zoonotic diseases, lack of religious dietary constraints, and significant collagen yields. In the dermal layers of fish, type I and V collagens predominate the fibrillar collagen. Despite the prevalence of type I collagen, the architectural and functional intricacies of type V collagen and its procollagen derivatives from marine origins have remained largely unexplored. This gap underlines the necessity of a thorough investigation into the structural and functional attributes of type V collagen derived from marine species, particularly large fish, to bolster scientific inquiry and application. The purpose of this study is to identify and characterize the structure of type V collagen in fish skin of shortbill spearfish, providing a basis for investigating the potential of type V collagen as a precursor for bioactive peptides.

## 2. Results and Discussion

### 2.1. Collagen Identification

**Figure 1:**
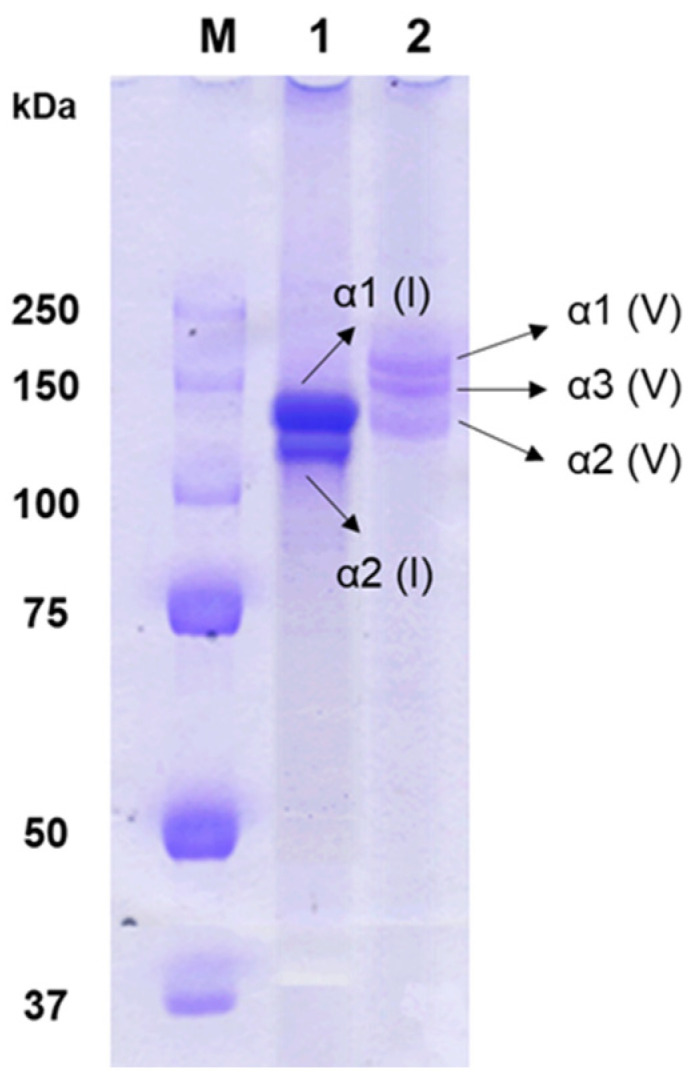
SDS-PAGE patterns of type I and V collagens isolated from shortbill spearfish. Lanes M, 1, and 2 indicate molecular weight markers, type I collagen, and type V collagen, respectively.

Figure 1 displays the SDS-PAGE pattern of type V collagen from the skin of shortbill spearfish. Type V collagen consisted of α1(V), α3(V), and α2(V) chains, aligning with its heterotrimeric structure [23]. In contrast, type I collagen included α2(I), along with 2α1(I) chains exhibiting twice the intensity of the α2(I) chain. The α3 chain in type I collagen could not be verified due to the overlapping electrophoretic migration position with those of α3(I) and α1(I) [24]. Furthermore, type I and V collagens isolated from shortbill spearfish demonstrated remarkable purity and structural integrity. This was prominently indicated by the absence of any other α subunits in the electrophoretic analyses. Under typical conditions, β and γ bands are visible in the SDS-PAGE patterns of collagens, suggesting the presence of cross-links between α chains. However, such bands were notably absent in the results obtained from these samples. The variability in the degree of collagen cross-linking observed among the samples may be attributable to seasonal variations in fishing practices [25,26]. Specifically, it has been observed that collagens extracted from fish that have endured periods of starvation show a higher degree of cross-linking compared to those derived from well-nourished counterparts, likely due to adaptive biological responses to nutritional stress [27].

Type V collagen consisted of α1(V), α3(V), and α2(V) chains with molecular weights of 154 kDa, 145 kDa, and 127 kDa, respectively, which were larger than those of corroborating findings of type I collagens with α1(I) and α2(I) chains with molecular weights of 130 kDa and 120 kDa, respectively, from prior studies on shortbill spearfish skin [28,29]. These results suggest that type V collagen has extensive intra- and/or intermolecular cross-linking, more than that of type I collagen. The SDS-PAGE pattern distinctly revealed three unique procollagens within type V collagen, laying the groundwork for subsequent investigations.

### 2.2. Physicochemical Properties of Shortbill Spearfish Type V Procollagens

Degenerate primers were constructed for the PCR reaction, which was conserved at the highest similarity among the nearest genetic relationship of bony fish α1(V), α2(V), and α3(V) procollagens. The sequence of obtained cDNA fragments was verified using the BLAST in the NCBI database. The cDNAs encoding Tacol5a1, Tacol5a2, and Tacol5a3 procollagens were cloned successfully. The sequence analysis showed that ORFs of cDNAs encoding Tacol5a1, Tacol5a2, and Tacol5a3 consisted of 5991, 4485, and 5607 bps, respectively, encoding 1997, 1495, and 1869 amino acid residues with a theoretical isoelectric point of 5.06, 6.75, and 5.76, respectively (Table 1). The calculated instability indexes of Tacol5a1, Tacol5a2, and Tacol5a3 procollagens were 32.61, 26.47, and 30.56, respectively, indicating that the procollagens are stable (Table 1). The determined nucleotide sequences of cDNAs encoding Tacol5a1, Tacol5a2, and Tacol5a3 procollagens have been deposited in the NCBI database under the accession numbers OR700193, OR700194, and OR700195, respectively.

**Figure 2:**
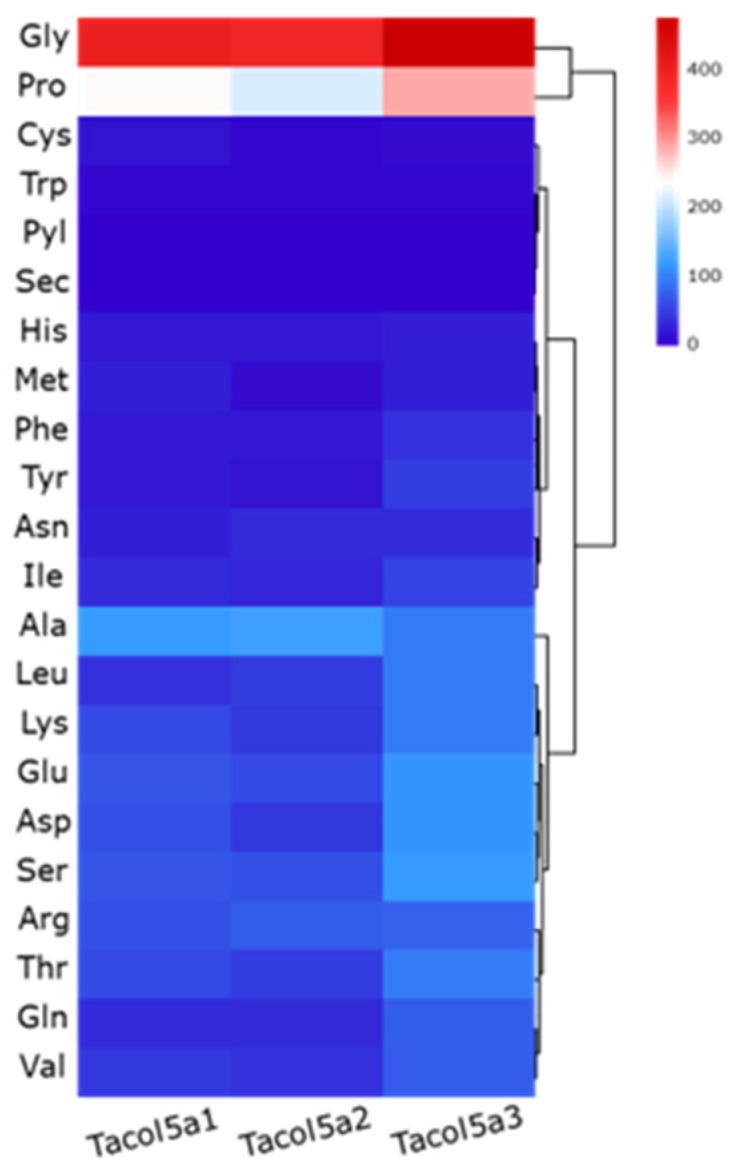
Heatmaps of deduced amino acids compositions of shortbill spearfish type V procollagens of Tacol5a1, Tacol5a2, and Tacol5a3.

The heatmaps of amino acid compositions of Tacol5a1, Tacol5a2, and Tacol5a3 procollagens are shown in Figure 2. In the compositions, glycine (Gly) was the most abundant (22–27%), followed by proline (Pro) (12–17%), indicating a rich GC content in the amino acid sequence of procollagens [30]. This typical structure of collagen chains determines the regular structure and contributes to the formation of stabilizing bonds [31]. Meanwhile, cysteine (Cys) and tryptophan (Trp), which are not supposed to be in the triple-helix structure [24], were present at less than 1%. The results are similar to the amino acid compositions of α-chains in type I and V collagens isolated from other species [31,32,33,34].

The contents of Gly-Pro-Pro (GPP) and Gly-Gly (GG) motifs in the triple-helical domains of shortbill spearfish Tacol5a1, Tacol5a2, and Tacol5a3 procollagens are shown in Table 2. The GPP motif contents in the triple-helical domains of shortbill spearfish type V procollagens of Tacol5a1, Tacol5a2, and Tacol5a3 (43, 35, and 31, respectively) were higher than those of shortbill spearfish type I procollagens of Tacol1a1 and Tacol1a2 (29 and 26, respectively). With the exception of the Tacol5a2 procollagen, aquatic animals contained the levels of the GPP motif lower than those of terrestrial animals. The content of the GPP motif was similar among animals from similar living environments. Total GPP motif content is the major factor influencing the thermal stability of collagen [35]. Estimating the Gly-Pro-Hyp content in mature proteins is feasible through analysis of the GPP content within the cDNA sequence, which could be used to predict the thermal stability of collagen [36,37]. Therefore, the higher GPP contents of shortbill spearfish type V procollagen indicated a more stable structure of type V collagen than type I collagen. This result is consistent with the thermal behavior reported by Wang et al. [29]. The Tacol5a1 procollagen showed the highest stability, which was associated with a high cross-linking of α1(V) [38]. However, the lower GPP content of shortbill spearfish type V procollagen compared with those of terrestrial animals indicates a lower denaturation temperature, which may be due to their cold-water habitat. On the other hand, the GG motif could be responsible for the partial skewing of the triple-helix structure and reduced thermal stability [32,37]. The lower GG motif content is also attributed to the higher thermal stability of shortbill spearfish type V collagen compared to zebrafish and sailfish.

### 2.3. Primary Structure Analysis of Shortbill Spearfish Type V Procollagens

**Figure 3:**
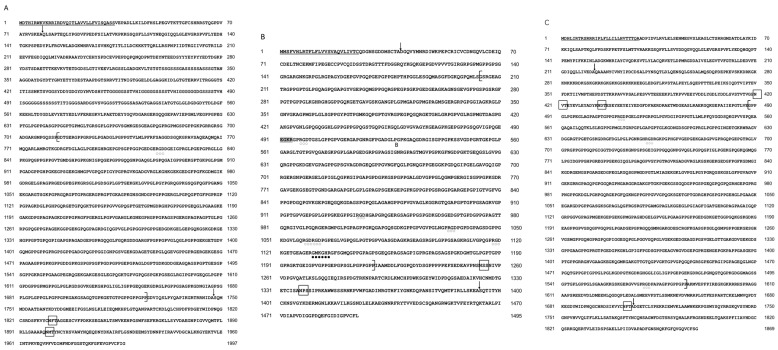
The deduced amino acid sequences of shortbill spearfish Tacol5a1 (**A**), Tacol5a2 (**B**), and Tacol5a3 procollagens (**C**). A single underlined letter indicates the cleavage site of the putative signal peptide, arrows indicate cleavage sites of N-propeptides and C-propeptides, and the putative intermolecular cross-linking sites are marked by black closed circles. The RGD sites that represent the potential cell adhesion sites are marked by open circles, Asn-X-Thr/Ser indicates glycosylation sites are boxed, and a shadow indicates the amidation site.

Regulation of mRNA transcription is critically influenced by the triple-helix structure [39]. The deduced amino acid sequences of Tacol5a1, Tacol5a2, and Tacol5a3 procollagens from shortbill spearfish are shown in Figure 3. Tacol5a2 procollagen contained one internal coupling site (GMKGHR). These interleaved coupling structures help stabilize the collagen fiber structure [40]. Furthermore, Tacol5a1, Tacol5a2, and Tacol5a3 procollagen contained 1, 6, and 4 arginine–glycine–aspartate (RGD) cell adhesion sites, respectively. It is noteworthy that potential Asn-X-Thr/Ser glycosylation sites are present in all of Tacol5a1, Tacol5a2, and Tacol5a3 procollagens (indicated by boxes). Among them, the glycosylation site of Tacol5a2 procollagen was found only in the C-terminal non-triple-helical structure. Tacol5a2 procollagen features an amidation reaction site (EGKR), a characteristic not widely conserved across different fish species. Consequently, while the amino acid sequence of shortbill spearfish type V procollagens shared similarities with those of other fish, notable distinctions were also present. Moreover, the putative N- and C-proteinase cleavage sites were shown, which were based on data from the other vertebrate procollagen chains [41]. In the N-terminus of Tacol5a1, Tacol5a2, and Tacol5a3 procollagens, the signal peptides with 33 amino acid residues (^1^MDTHIRWKVKRRIRDVQITLAVVLLFVISQASS^33^), 25 amino acid residues (^1^MMS-FVHLRTFLFLVVSVAQVLIVTC^25^), and 28 amino acid residues (^1^MDHLIRTRSRRRIPLFLLI-LLHVTTTQA^28^) were observed, respectively.

**Figure 4:**
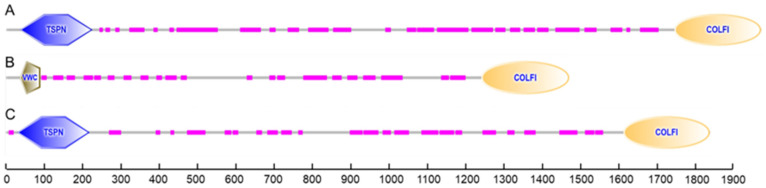
The prediction of conserved domains of shortbill spearfish Tacol5a1 (**A**), Tacol5a2 (**B**), and Tacol5a3 (**C**) procollagens.

According to the identification of conserved domains, each of the deduced amino acid sequences of procollagens contained a conservative domain of the fibrillar collagen C-terminus (COLFI), suggesting that shortbill spearfish type V collagens produced from Tacol5a1, Tacol5a2, and Tacol5a3 procollagens are members of the fibrillar collagen family (Figure 4). Moreover, the thrombospondin-like N-terminal domains (TSPN) related to the heparin-binding and cell adhesion domain of thrombospondin were found in Tacol5a1 and Tacol5a3 procollagens. The presence of TSPN suggested the potential of Tacol5a1 and Tacol5a3 procollagens to influence the physiology and pathology of cardiovascular disease [13]. Furthermore, the von Willebrand factor (VWC) domains [42] were observed at the N-terminus of Tacol5a2 procollagen, potentially suggesting an impact on platelet function.

### 2.4. Secondary and Tertiary Structure Prediction of Shortbill Spearfish Type V Collagens

**Figure 5:**
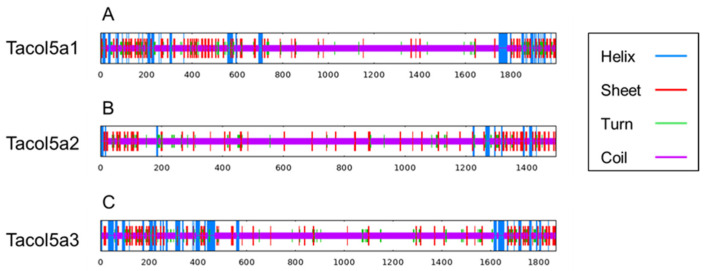
The prediction of the secondary structure of shortbill spearfish Tacol5a1 (**A**), Tacol5a2 (**B**), and Tacol5a3 (**C**) procollagens. The blue line represents the α-helix, the red line represents the extended strand, and the purple line represents the random coil.

As shown in Figure 5 and Table 3, the contents of α-helix, β-sheet, and turn are concentrated at the N- and C-termini of shortbill spearfish type V procollagens. Tacol5a1 procollagen showed the highest beta sheet content (13.32%), and lower coil content (71.06%), indicating the higher stability. These results are consistent with the previous discussion of the GPP motif content, which could account for the stable structural stability. Furthermore, alpha helix and beta sheet were mainly distributed in the C- and N-termini of procollagens, and most of the triple-helix domains were coiled, which is also a special characterization of collagen structure.

**Figure 6:**
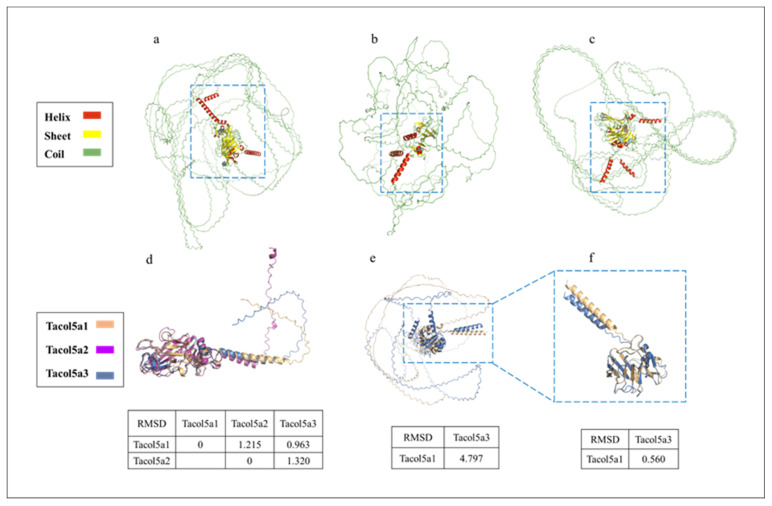
Tertiary structure prediction of shortbill spearfish Tacol5a1, Tacol5a2, and Tacol5a3 procollagens. Tertiary structure prediction of Tacol5a1, Tacol5a2, and Tacol5a3 procollagens are shown in panels (**a**–**c**); the C-terminus and N-terminus without triple-helix structures are boxed; (**d**) indicates an alignment of the tertiary structure of the C-terminus; (**e**) denotes an alignment of the tertiary structure of Tacol5a1 and Tacol5a2 procollagens without triple-helix structural domains; (**f**) denotes an alignment of the structure of the N-terminus without telopeptide.

The tertiary structure prediction of shortbill spearfish procollagens by AlphaFold 2 based on Colab is shown in Figure 6, suggesting that the procollagens are spheroidal (Figure 6a–c). Hence, the Colab-based AlphaFold2 could not predict the 3D structure very accurately for collagen. Figure 6d,e show the C- and N-terminal structural domain comparisons, respectively. Quantitative assessment of similarity between two protein structures was achieved by calculating RMSD after superimposing them in PyMOL software. In Figure 6d, the RMSD values between the two procollagens were small (<1.35), indicating a high level of structural similarity [43]. Further, since no conserved domains were found at the N-terminus of Tacol5a2 procollagen in Figure 4, only the N-terminal region without the triple-helix domain of Tacol5a1 and Tacol5a3 procollagens are shown in Figure 6e. The RMSD level of the structures of Tacol5a3 and Tacol5a1 procollagens was 4.797, indicating a structural similarity of less than 50% [44]. However, the removal of the N-terminal telopeptide reduced the RMSD level to 0.560, indicating the structure similarity is higher than 80% (Figure 6f). These results suggest that Colab-based AlphaFold2 still needs further deep learning on the tertiary organization prediction of proteins with high coil content and a special structure.

### 2.5. Multiple Sequence Alignment and Phylogenetic Analysis

**Figure 7:**
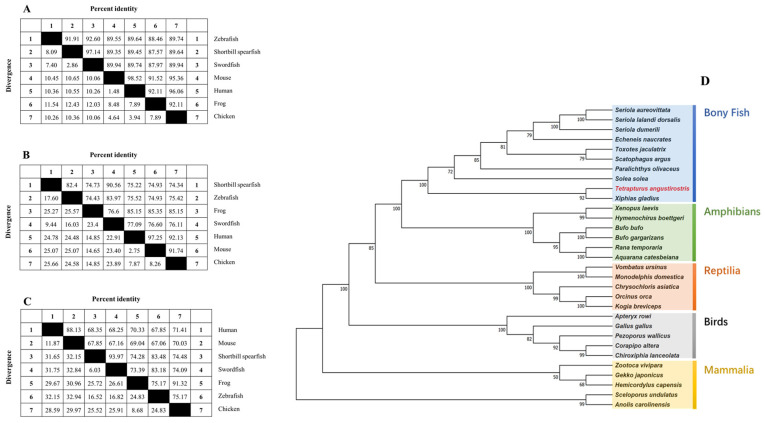
The homology of shortbill spearfish Tacol5a1 (**A**), Tacol5a2 (**B**), and Tacol5a3 (**C**) procollagens with other procollagens and their phylogenetic relationship (**D**). (**D**) is a maximum likelihood (ML) tree based on the amino acid sequences of procollagens. The other amino acid sequences were downloaded from the NCBI protein database. The phylogenetic tree was constructed by joining amino acid sequences of various species. Numbers at nodes indicate bootstrap values for 1000 replicates.

Figure 7 presents the comparative homology of amino acid sequences for procollagens across various species, revealing significant conservation between shortbill spearfish procollagens and those from other species. Figure 7A–C illustrate that the degree of homology for type V procollagens varies across species, with Tacol5a1 procollagen demonstrating the most substantial sequence conservation. Notably, the identities between all three shortbill spearfish procollagens and that of broadbill swordfish exceed 90%. Specifically, the identities are approximately 89% for Tacol5a1 procollagen, 74% to 83% for Tacol5a2 procollagen, and 67% to 84% for Tacol5a3 procollagen. Remarkably, Tacol5a1, Tacol5a2, and Tacol5a3 procollagens shared a high degree of identities with broadbill swordfish (97.14%, 90.56%, and 93.97%, respectively).

Additionally, the phylogenetic tree of shortbill spearfish is shown in Figure 7D, which was constructed via a multi-locus sequence analysis. All examined sequences were clustered into Col5a1, Col5a1, or Col5a3, with a topology that aligned with the established phylogeny of bony fish. Notably, the closest homology observed was between shortbill spearfish and broadbill swordfish, highlighting a distinct evolutionary lineage among bony fish, amphibians, reptiles, birds, and mammals.

### 2.6. Potential Bioactivity of Type V Collagen by In Silico Method

**Figure 8:**
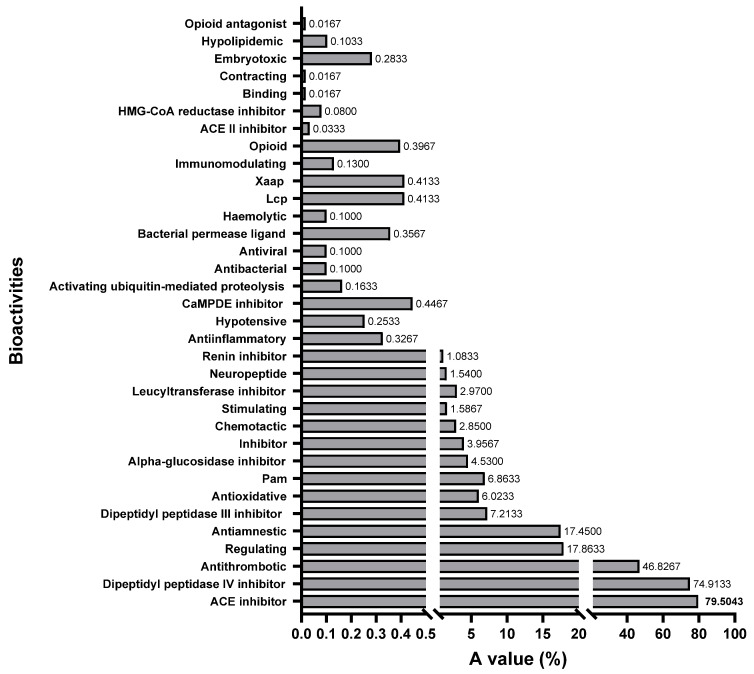
The frequency of occurrence of peptides with a given activity (A value) in type V collagen of shortbill spearfish.

All the bioactivities in the database were selected to evaluate the feasibility of the three shortbill spearfish type V procollagens all together as a precursor for bioactive peptides by comparing the A values [45]. The A value denotes the frequency of occurrence of the bioactive fragments in collagen. A higher A value represents the higher potential of the protein to produce bioactive fragments. The frequency of occurrence for bioactive fragments in each bioactive type is shown in Figure 8. It is evident from this figure that fragments with dipeptidyl peptidase IV inhibition activity (74.91%), ACE inhibitory activity (75.90%), and thrombin inhibitory activity (46.83%) dominate the sequences of all type V procollagens. This suggests a significant presence of these activities within the collagen sequence. The BIOPEP-UWM database contains 48 major types of peptide bioactivities, of which 34 subclasses of peptide bioactivities could be identified in the obtained type V collagen amino acid sequence, indicating that type V collagen is a valuable potential candidate for the production of bioactive peptides.

## 3. Materials and Methods

### 3.1. Materials

Shortbill spearfish specimens (body weight: 12–18 kg) were purchased from fishermen in Kesen-Numa City, Miyagi Prefecture, Japan. They were frozen immediately after being caught and transported to the laboratory of Tokyo University of Marine Science and Technology. Pepsin (1:10,000) (from Porcine Stomach Mucosa, EC 3.4.23.1), NaOH, acetic acid, HCl, NaCl, tris-(hydroxymethyl)-aminomethane (Tris), butanol, and bromophenol blue were purchased from FUJIFILM Wako Pure Chemical Industries, Ltd. (Osaka, Japan). Ultrapure water was prepared by Milli-Q system (Millipore, Tokyo, Japan).

### 3.2. Fish Skin Pretreatment

The fish skin was washed after removing the flesh and cut into small pieces (less than 0.5 × 0.5 cm^2^). A solution of 0.1 M of NaOH was used to soak the skin at a ratio of 1:30 (*w*/*v*) at 4 °C for 48 h in order to remove the non-collagenous protein, refreshing the alkaline medium solution every 6 h. The skin pieces were cleaned to a neutral pH with cold water, followed by treatment with 10% butanol. Concurrently, non-collagenous proteins and alkaline-soluble collagens were eliminated, and the remaining materials were rinsed with cold ultrapure water until it reached a neutral to slightly basic pH level. The defatted skin was cleaned and stored at −25 °C. Before use, the skin was chilled at −85 °C for 6 h and then minced with a grinder (SKF-H100, Tiger Magic Bottle Co., Ltd., Osaka, Japan). All steps were conducted at 4 °C, with continuous agitation using a magnetic stirrer.

### 3.3. Preparation of Type V Collagen

The extraction of distinct collagen types was performed using the method described by Han et al. [28]. The pretreated skin was agitated in 0.5 M acetic acid containing 0.1% pepsin (*w*/*v*) at a 1:20 (*w*/*v*) ratio for 48 h, then centrifuged at 10,000× *g* for 60 min. The supernatant underwent salting out by the addition of NaCl to achieve a final concentration of 1.2 M, followed by centrifugation at 10,000× *g* for 60 min. The resulting precipitate was redissolved in 0.5 M Tris-HCl buffer (pH 7.5), and the NaCl concentration was subsequently adjusted to 4.0 M. The precipitate was redissolved and then added with 2.4 M NaCl to separate the type V and type I collagens into supernatant and precipitate fractions, respectively. The supernatant was dialyzed against ultrapure water with dialysis membranes (MWCO:12–14 kDa, standard RC tubing, Repligen Corp., Waltham, MA, USA). The precipitate was dissolved into 0.5 M acetic acid and dialyzed against 0.1M acetic acid and ultrapure water, successively. The pepsin-soluble collagen solutions were stored at −25 °C after lyophilization. All procedures were performed at 4 °C.

### 3.4. SDS–Polyacrylamide Gel Electrophoresis (SDS-PAGE) Pattern

SDS-PAGE of pepsin-soluble collagen extracted from the fish skin was performed according to the method of Laemmli [46] with some modifications. A sample at 2 mg/mL in 0.5 M acetic acid was treated with 2× sample loading buffer (60 mM Tris–HCl, pH 8.0, containing 25% glycerol, 2% SDS, 0.1% bromophenol blue) in the presence of 2% β-mercaptoethanol. SDS-PAGE of pepsin-soluble collagen was conducted on 8% resolving gel and 5% stacking gel on a cPAGE Ace Twin (WSE-1025W, ATTO Co., Ltd., Tokyo, Japan). After electrophoresis, the gel was stained with 0.1% (*w*/*v*) Coomassie blue R-250 and then destained.

### 3.5. cDNA Cloning of Procollagens

#### 3.5.1. RNA Extraction and cDNA Synthesis

The skin was dissected out from shortbill spearfish, cut into small pieces, and transformed into liquid nitrogen immediately. Frozen tissues were put into a 50 mL tube and homogenized with TRIzol™ Reagent (Invitrogen, Carlsbad, CA, USA) to isolate total RNA. Poly A^+^ RNA was isolated and purified from total RNA using illustra™ QuickPrep Micro mRNA Purification Kit (GE Healthcare, Tokyo, Japan) according to the manufacturer’s instructions. The quality, purity, and integrity of RNA were assessed by the A260/280 ratio.

Synthesis of double-stranded cDNA (ds cDNA) was performed with Marathon^®^ cDNA Amplification Kit (Clontech Lab, Mountain View, CA, USA) according to the manufacturer’s introductions.

#### 3.5.2. Cloning of cDNA Encoding Procollagens

The gene-specific primers (GSPs) of procollagens nucleotide sequences (Tacol5a1, Tacol5a2, and Tacol5a3) were constructed based on the conserved regions selected from broadbill swordfish *Xiphias gladius* (XM040158199.1, XM040148102.1, XM040157418.1), mandarin fish (*Siniperca chuatsi*) (XM044175423.1, XM044215955.1, XM044180658.1), redfin perch (*Perca fluviatilis*) (XM039779366.1, XM039817710.1, XM039825796.1), leopard coral grouper (*Plectropomus leopardus*) (XM042508952.1, XM042494419.1, XM042504858.1), yellowtail amberjack Seriola (*lalandi dorsalis*) (XM023422226.1, XM023423150.1, XM023401417.1), barramundi perch (*Lates calcarifer*) (XM051072972.1, XM018683186.2, XM018705093.2), and banded archerfish (*Toxotes jaculatrix*) (XM041058269.1, XM041056572.1, XM041061690.1) using the ClustalW program (https://www.genome.jp/tools-bin/clustalw, accessed on 19 March 2023) (Supplementary Materials).

The cDNA sequences encoding Tacol5a1, Tacol5a2, and Tacol5a3 were obtained using the template diluted 250 times from the adapted ds cDNA. The PCR reaction was carried out in a total volume of 25 µL using Ex Taq DNA polymerase (Takara, Otsu, Japan). PCR thermal cycling conditions were 30 cycles of 98 °C for 10 sec for denaturation, 53–55 °C 30 sec for annealing, and 72 °C for 1 sec for extension, with a final extension step at 72 °C for 3 min. The detailed annealing temperature depended on each primer pair. The cDNAs of Tacol5a1, Tacol5a2, and Tacol5a3 were obtained using the sets of adaptor primers (AP1: CCATCCTAATACGACTCACTATAGGGC, AP2: ACTCACTATAGGGCTCGAGCGGC) with GSP-AR (5′-end) or GSP-AF (3′-end) (Supplementary Materials). PCR products were gel-purified by FastGene™ Gel/PCR Extraction Kit (Nippon Gene, Tokyo, Japan), subcloned into the pGEM-T easy vector (Promega, Masison, WI, USA), treated with a BigDye Terminator V3.1 Cycle Sequencing Kit (Applied Biosystems, Foster City, CA, USA), and sequenced with an ABI 3130 Genetic Analyzer (Applied Biosystems). The cDNA sequences of Tacol5a1, Tacol5a2, and Tacol5a3 were generated by overlapping the fragments.

### 3.6. Bioinformatics Analysis

The cDNA sequences and deduced amino acid sequences were analyzed using SnapGene software (www.snapgene.com, accessed on 1 April 2023). The open reading frames (ORFs) were identified using the NCBI ORF Finder (http://www.ncbi.nlm.nih.gov/projects/gorf/, accessed on 1 August 2023). Sequence similarity was explored using the BLAST tool (http://blast.ncbi.nlm.nih.gov/, accessed on 1 August 2023). Physical and chemical parameter calculations were performed using the Expasy-ProtParam tool (https://web.expasy.org/protparam/, accessed on 5 August 2023). Secondary structure prediction was carried out using ESPript V3.0 (https://espript.ibcp.fr/ESPript/ESPript/, accessed on 7 August 2023). The prediction of conserved domains in the amino acid sequence was performed using the SMART program (http://smart.embl-heidelberg.de/, accessed on 7 August 2023). The signal peptides were determined using the software SignalP 4.0 (https://services.healthtech.dtu.dk/services/SignalP-4.1/, accessed on 8 August 2023). ColabFold V1.4 was used to model the tertiary structure of Tacol5a1, Tacol5a2, and Tacol5a3 procollagens [47]. To quantitatively assess the similarity between two protein structures, the root mean square deviation (RMSD) was calculated by superimposing the structures using PyMOL Molecular Graphics System v2.4.0 (Schrodinger, LLC.; New York, NY, USA) [48]. A phylogenetic neighbor-joining tree was constructed using Mega11 V11.0.13 [49], and 1000 bootstrap trials were conducted to increase the confidence values for the resulting phylogenetic tree.

### 3.7. Assessment of Bioactive Peptides in Type V Collagen

The assessment of potential bioactive peptides in type V collagen was carried out using BIOPEP-UWM analysis (https://biochemia.uwm.edu.pl/en/biopep-uwm-2/, visited on January 2024) [50]. The amino acid sequence of each procollagen chain was subjected to “profiles of potential biological activity”. The potential bioactive peptides from type V collagen derived from shortbill spearfish were screened. The frequency of fragments with bioactivities in protein sequences, denoted as A%, was characterized using the following equation:

$$A(\%)=\frac{a}{N}\times 100$$

where a is the number of fragments and N is the number of amino acid residues of the protein.

## 4. Conclusions

This study marks the cloning of cDNAs encoding type V procollagen from shortbill spearfish skin. The findings highlight Tacol5a1, Tacol5a2, and Tacol5a3 procollagens for exceptional thermal stability. Analysis of the deduced amino acid sequences revealed that shortbill spearfish type V collagens shared fundamental biological functions with those in other bony fish (more than 90%). Additionally, *in silico* analysis confirmed the potential of shortbill spearfish type V collagens to generate bioactive fragments. The investigation of the bioactivity of shortbill spearfish type V collagens is the forthcoming work in our research. Improving the accuracy of collagen tertiary structure prediction will also be a future research goal.

## Figures and Tables

**Table 1:** Physicochemical properties of shortbill spearfish Tacol5a1, Tacol5a2, and Tacol5a3 procollagens.

Encoding Gene Name	Amino Acid Residues	Molecular Mass (kDa)	pI	Instability Index
Tacol5a1	1997	198.44	5.06	32.61
Tacol5a2	1495	145.06	6.75	26.47
Tacol5a3	1869	189.15	5.76	30.56

**Table 2:** Gly-Pro-Pro and Gly-Gly contents in the triple helical domain of shortbill spearfish Tacol5a1, Tacol5a2, and Tacol5a3 procollagens in comparison with those of other animals.

	Triple Helix Region Length	Gly-Pro-Pro Content	Gly-Gly Content	Accession Number
α1(I) and α2(I) procollagens				
Shortbill spearfish Tacol1a1	1014	29	13	OR700191
Shortbill spearfish Tacol1a2	1015	26	14	OR700192
α1(V) procollagen				
Shortbill spearfish Tacol5a1	1014	43	4	
Zebrafish	1014	42	3	ADG36303.1
Broadbill Swordfish	1014	43	6	XP040014133.1
Human	1014	49	7	NP000084.3
Mouse	1014	44	7	EDL08374.1
Chicken	1014	48	3	NP990121.2
Shortbill spearfish α2(V) procollagen				
Tacol5a2	1017	35	6	
Zebrafish	1017	34	11	NP001139254.1
Broadbill Swordfish	1017	32	4	XP040004036.1
Human	1017	30	6	NP000384.2
Mouse	1017	30	6	NP031763.2
Chicken	1017	30	11	XP040532372.1
α3(V) procollagen				
Shortbill spearfish Tacol5a3	1011	31	11	
Zebrafish	1011	32	11	NP001177685.1
Broadbill Swordfish	1011	30	10	XP_040013352.1
Human	1011	41	4	NP001845.3
Mouse	1011	43	5	AAF59901.1
Chicken	1011	40	6	XP422303.4

**Table 3:** Prediction of secondary structure composition of shortbill spearfish Tacol5a1, Tacol5a2, and Tacol5a3 procollagens.

%	Alpha Helix	Beta Sheet	Turn	Coil
Tacol5a1	9.81	13.32	5.81	71.06
Tacol5a2	3.81	11.64	4.75	79.80
Tacol5a3	12.52	11.56	6.37	69.56

## Data Availability

No new data were created or analyzed in this study. Data sharing is not applicable to this article.

## Supplementary Materials

The following supporting information can be downloaded at: https://www.mdpi.com/article/10.3390/molecules29112518/s1, Table S1: Primer names and sequence information. Figure S1: Primers location at each coding regions of shortbill spearfish procollagens.
