# Supplementary material for: Functional and Structural Properties of Type V Collagen from the Skin of the Shortbill Spearfish (Tetrapturus angustirostris)
Source: Molecules. 2024 May 27;29(11):2518. doi: 10.3390/molecules29112518 (PMC11173678; doi:10.3390/molecules29112518)
Supplement: Supplementary file 1 [file molecules-29-02518-s001.zip › molecules-3014428-supplementary.pdf]

Table S1 Primer names and sequence information

Primer name	Sequence
1a1-F1	TGACTGGTAGCCCTGGAAG
1a1-R1	AGGAGGTCCAGCGAATCC
1a1-F2	GGAGCTGCTGGCCCTCAGGGTGC
1a1-R2	CCTCTCTCACCAGGCTTACC
1a1-F3	TGGTGAGGCTGGAGAGAGA
1a1-R3	GAACCAGACGTGCTTCTTCTC
1a1-F4	TCCTGGTGCTACTGGTTANCC
1a1-R4	TCTCTCTCCAGCCTCACCA
1a1-F5	ATGTTCAGCTTTGTGGATATTCG
1a1-R5	GCTCCATCCATACCACTGAATC
1a1-F6	TGACTGGTAGCCCTGGAAG
1a1-R6	AGGAGGTCCAGCGAATCC
1a1-F7	GAGWCAGAGGAGAGGCTGGT
1a1-R7	CGCTGGGGTAGATGCAAGT
1a2-F3	GTGGTGAGAAGGGAGKTGCTG
1a2-R3	GTTCTTGCGGGCAATGCT
1a2-F4	AGCAGGGTATNGTTGGACCAC
1a2-R4	CAGSMACTCCCTTCTCACCAC
1a2-F5	GATGGTGGCAAGGGAGAG
1a2-R5	GTGGTCCAANCATACCCTGCT
1a2-F6	TGGAGATCCTGGTNNTCAG
1a2-R6	CTCTCCCTTGCCACCATC
1a2-F7	GGTTACACTGGTCTGGATGGAC
1a2-R7	TCCTCTCTTGCCCTCCTC
1a2-F8	GTCCATCCAGACCAGTGTAACC
1a2-R8	GTCCATCCAGACCAGTGTAACC
5a1-F1	GGATAAGGGACGCCCAAATCACT
5a1-R1	CATAATAGTAGCTGTCCTCCTCTGTG
5a1-F2	CACAGAGGAGGACAGCTACTATTATG
5a1-R2	ATGGTAGAACTGCTGCTGCT
5a1-F3	CTGAAGGGAGAGTCTGGAGAG
5a1-R3	GTCCTTTAAGACCACGAACACC
5a1-F4	CACTTTAGCGGTGGTTCTGC
5a1-R4	GTAGTGCTCACAGTAGTCATAGGC
5a1-F5	GAAGGGAGAGCCTGCTGTT
5a1-R5	CCATCCAACCCTCKGTCTCC
5a1-F6	GGAGACNGAGGGTTTGATGG
5a1-R6	CCTCNGGACCATCTTCTCC
5a1-F7	CTGGAGANGTTGGTCANATGG
5a1-R7	GAGTCTCCTTTCAAACCNGGT
5a1-F8	AAGGATCACCTGGCAAGC
5a1-R8	GCCAAAGATTTCCTCCATGC

5a2-F3	ACATGTCAAGANGGGAACAG
5a2-R3	GATCCAGGGAATCCAACTTC
5a2-F4	GAAGTTGGATTCCCTGGATC
5a2-R4	AGTGCTTCCCTGAGGTCCTG
5a2-F5	AGGACCTCAGGGAAGCACT
5a2-R5	GGAATCCATTTACACCTGGAG
5a2-F6	TNGCAGGAGACAGAGGAGAG
5a2-R6	GCCTTGNGGTCCTGGTGAT
5a2-F7	CAACCYGGAGTCAAGGGAGA
5a2-R7	TCTCCTCTCNTTCCAGCATCACC
5a2-F8	GGTGATGCTGGAANGAGAGGAG
5a2-R8	CACCACACTTTACGAGGTATGC
5a2-F9	CACTGCAACATGGACACC
5a2-R9	TTACAAGAAGCACACNGGCC
5a3-F1	CTGGAGCTGTCAGAGAACAT
5a3-R1	CAGACCCAAGTTGNGAGTTC
5a3-F2	TGAGGGAGATATTCAGCAACTACT
5a3-R2	TCTCCCTTCTGGCCTTTCTC
5a3-F3	GAGAAAGGCCAGAAGGGAGAG
5a3-R3	CCAGGCTTTCCTCTGTCC
5a3-F4	CCAGGTGAAACAGGCTCTAAGG
5a3-R4	GCCATCTTCTCCCTTCTCTCCT
5a3-F5	GATACCCAGGAAGACAAGGTNC
5a3-R5	CTCCTTTGTCTCCTTTGCTG
5a3-F6	GCAGCAAAGGAGACAAAGG
5a3-R6	CATGCCTTGCTTCCCTTC
5a3-F7	GGGAAGCAAGGCATGAAG
5a3-R7	GACTTCTTCCATTCCCTCNG
5a3-F8	CNGAGGGAATGGAAGAAGTC
5a3-R8	AACCNNTGAAGCATACTCGTC
1a1-AR1	CGCATATCCACAAAGCTGAAC
1a1-AF1	CAAGAGTTTGGTGTGGACATCGGCCCTGTT
1a2-AR1	GCTAGGTATGAAGTTACTGCAAGC
1a2-AF1	AGGAATTCGGATTGGACATCGGCCCTGTCT
5a1-AR1	AAGTGATTTGGGCGTCCCTTATCC
5a1-AF1	GCCAAAGATTTCCTCCATGC
5a2-AR1	CCTTAAATGCACAAAGCTCATC
5a2-AF1	GGCCCGTGTGCTTCTTGTAA
5a3-AR1	TGGGAGCACTGAGCTGGATCTTT
5a3-AF1	CTGCTCCCTATTATAGATGTGGC

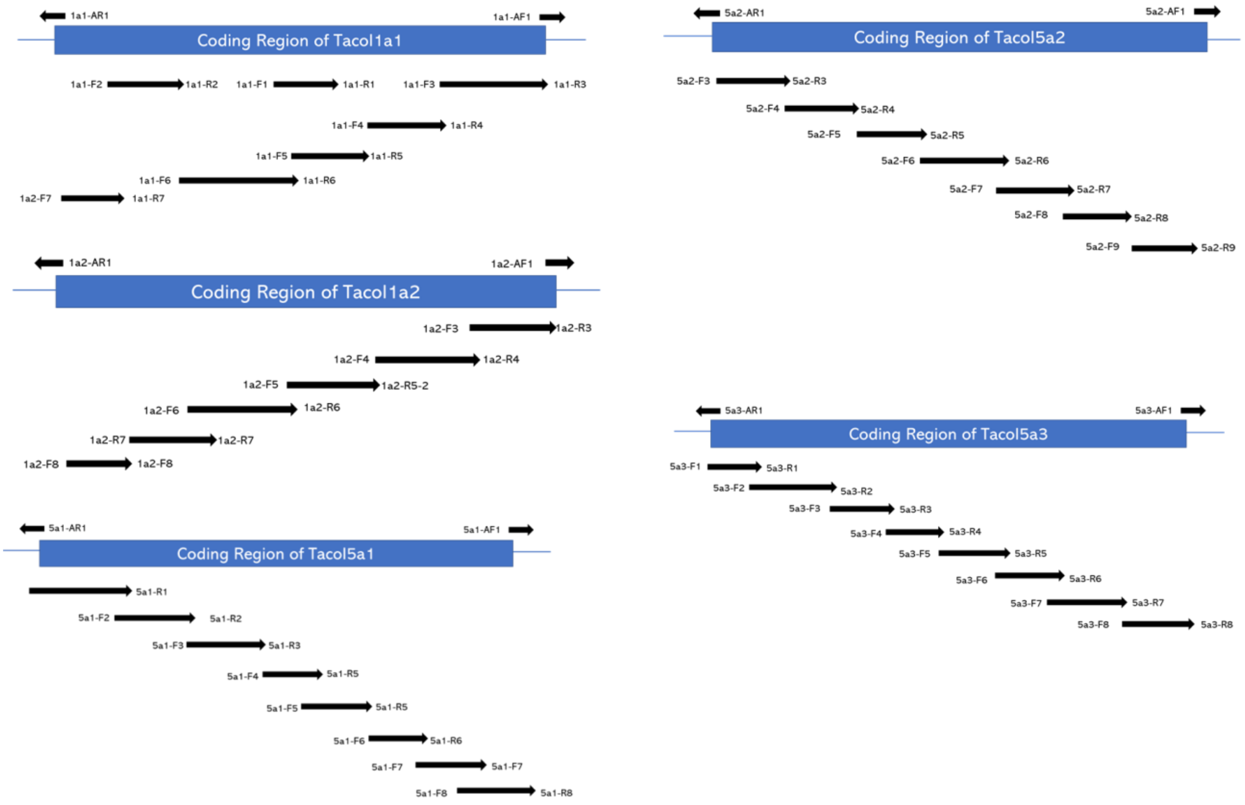

Figure S1 Primers location at each coding regions of shortbill spearfish procollagens.
